# Recommended intakes of vitamin D to optimise health, associated circulating 25-hydroxyvitamin D concentrations, and dosing regimens to treat deficiency: workshop report and overview of current literature

**DOI:** 10.1017/jns.2015.10

**Published:** 2015-05-25

**Authors:** Michiel G. J. Balvers, Elske M. Brouwer-Brolsma, Silvia Endenburg, Lisette C. P. G. M. de Groot, Frans J. Kok, Jacqueline Klein Gunnewiek

**Affiliations:** 1 Clinical Chemistry and Haematology Laboratory, Gelderse Vallei Hospital, PO Box 9025, 6710 HN, Ede, the Netherlands; 2 Division of Human Nutrition, Wageningen University, PO Box 8129, 6700 EV Wageningen, the Netherlands

**Keywords:** Vitamin D, Workshop reports, Optimum intake, Deficiency, 25(OH)D, 25-hydroxyvitamin D, BMD, bone mineral density, IOM, Institute of Medicine, IU, international unit, RCT, randomised clinical trial

## Abstract

Vitamin D is a fat-soluble hormone that traditionally has been linked to bone health. Recently, its involvement has been extended to other (extra-skeletal) disease areas, such as cancer, CVD, energy metabolism and autoimmune diseases. Vitamin D deficiency is a worldwide problem, and several recommendation-setting bodies have published guidelines for adequate vitamin D intake and status. However, recommendations from, for example, the Health Council of the Netherlands do not provide advice on how to treat vitamin D deficiency, a condition that is often encountered in the clinic. In addition, these recommendations provide guidelines for the maintenance of ‘minimum levels’, and do not advise on ‘optimum levels’ of vitamin D intake/status to further improve health. The NutriProfiel project, a collaboration between the Gelderse Vallei Hospital (Ede, the Netherlands) and the Division of Human Nutrition of Wageningen University (Wageningen, the Netherlands), was initiated to formulate a protocol for the treatment of vitamin deficiency and for the maintenance of optimal vitamin D status. To discuss the controversies around treatment of deficiency and optimal vitamin D status and intakes, a workshop meeting was organised with clinicians, scientists and dietitians. In addition, a literature review was conducted to collect recent information on optimal intake of vitamins, their optimal circulating concentrations, and effective dosing regimens to treat deficiency. This information has been translated into the NutriProfiel advice, which is outlined in this article.

## 1. Introduction

Vitamin D is primarily obtained via exposure to UV light, which initiates vitamin D production in the skin. In addition, vitamin D can also be acquired through the diet. However, there are only a few natural food sources – fatty fish, meat, eggs, whole dairy products – and in the Netherlands only a limited number of foods are enriched with vitamin D, like fats, margarines and spreads^(^^1^^,^^2^^)^. Two types of vitamin D exist: vitamin D_2_, which is plant derived; and the animal-derived vitamin D_3_. Most of the data presented in this paper focus on vitamin D_3_ (also known as cholecalciferol) because it is generally accepted that vitamin D_3_ is more effective than vitamin D_2_^(^^3^^)^.

Traditionally, vitamin D has been linked to bone health, and most of the randomised clinical trials (RCT) have focused on bone mineralisation and fracture risk^(^^1^^–^^3^^)^. However, in recent years, observational studies have revealed an inverse association between vitamin D status and the risk of cancer, diabetes, cognitive decline and certain autoimmune diseases^(^^1^^)^. Despite the wealth of publications reporting on associations between vitamin D and these health outcomes, there is not yet consensus on optimal intakes of vitamin D and reference concentrations of its metabolite, 25-hydroxyvitamin D (25(OH)D), the widely accepted vitamin D status indicator^(^^1^^)^. This is partly due to the lack of RCT-based data for areas other than bone health^(^^1^^,^^3^^)^. In addition, the lack of guidelines to treat deficiency and the controversy around ‘optimal’ over ‘minimum’ vitamin D status has further fueled discussions in this field. Several recommendation-setting bodies have published vitamin D recommendations in recent years, such as the Institute of Medicine (IOM)^(^^2^^)^, the Scientific Advisory Council on Nutrition^(^^4^^)^ (update in preparation), the Health Council of the Netherlands^(^^3^^)^ and the Nordic Council of Ministers^(^^5^^)^. Most of these recommendations have set target 25(OH)D values of 30 or 50 nmol/l (summarised in Brouwer-Brolsma *et al.*^(^^1^^)^), which have been heavily debated by vitamin D experts who proposed higher target values^(^^6^^–^^10^^)^. These controversies have led to widespread diversity regarding the treatment of vitamin D deficiencies. At the same time, it has become clear that the incidence of vitamin D deficiency is rising in Northern Europe^(^^11^^)^, and increased hospitalisation rates for deficiency-related disorders, such as rickets in children, have been reported^(^^12^^)^. In its 2012 recommendations, the Health Council of the Netherlands summarised studies that explored the prevalence of vitamin D deficiency within the Dutch population, demonstrating that significant differences exist between different ethnic groups^(^^3^^)^. For instance, within pregnant women, the prevalence of vitamin D deficiency ranged from 8 % among women with a Dutch background to 78 % among women with a Turkish background. A similar picture emerged in adult men and women; vitamin D deficiency was found in approximately 10 % of adults with a Dutch background, and in approximately 40 % of adults with a Surinam background^(^^3^^)^. This indicates that vitamin D deficiencies are widely present in Northern European countries, and it is clear that current strategies need to be revised in order to improve vitamin D status.

In order to bridge this gap, the Clinical Chemistry and Haematology Laboratory of the Gelderse Vallei Hospital, together with the Division of Human Nutrition of Wageningen University, initiated NutriProfiel. NutriProfiel aims to provide advice to treat vitamin deficiency and subsequently maintain optimal vitamin status. In June 2013, a workshop meeting was organised to discuss the controversies around optimal vitamin D status and the treatment of deficiencies. The meeting was attended by participants with a variety of backgrounds, including dietitians, clinicians and nutrition scientists (see Appendix). In addition to the workshop meeting, a literature survey was performed to collect recent information on optimal vitamin D status and intakes, and the treatment of deficiency. Particular attention was given to recommendations of the IOM^(^^2^^)^ and the Health Council of the Netherlands^(^^3^^)^. Attention was also given to publications proposing different cut-off values and intakes related to deficiency and sufficiency for bone health. The present paper summarises the main conclusions from the workshop meeting and the literature survey. This paper combines strategies to treat vitamin D deficiency with a critical discussion of current recommendations, leading to the NutriProfiel recommendations. The vitamin D recommendations from the Health Council of the Netherlands served as the basis for this.

Optimal 25(OH)D concentrations and vitamin D intakes to maintain optimal levels are defined (section 2), followed by the introduction of a strategy to treat vitamin D deficiency (section 3). This document also discusses potential safety issues (section 4), and briefly explains differences between the NutriProfiel advice and the Netherlands Health Council advice (section 5). Finally, the recommendations are summarised (section 6).

## 2. Optimal vitamin D intakes and 25-hydroxyvitamin D levels

Vitamin D is traditionally linked to effects on bone health, for example, reducing fracture risk and increasing bone mineral density (BMD)^(^^3^^)^. Effects on other systems, such as the cardiovascular system, pancreas or immune system, are also reported in the literature, but this evidence is usually derived from observational studies, in which causality cannot be examined^(^^1^^,^^3^^)^. Most of the intervention studies have focused on bone health, and therefore this section is limited to this area^(^^3^^)^. In contrast to the Health Council of the Netherlands, that has advised on minimum levels, NutriProfiel aims to provide recommendations concerning optimal levels. In this section, we provide an overview of evidence to support recommendations for optimal levels of vitamin D status, and the intakes necessary to maintain this optimal status; treatment of deficiency will be discussed in section 3.

### 2.1. Data for > 65-year-olds

Several RCT have been performed, using diverse study populations, different doses of vitamin D with or without additional Ca, different duration of supplementation and study outcome parameters. The studies have reported inconsistent conclusions concerning the relationship between vitamin D, 25(OH)D concentrations and fracture risk, i.e. either no effect of vitamin D, or a protective effect on fracture risk. Accordingly, meta-analyses have also reported inconsistent conclusions^(^^13^^–^^18^^)^, even when relatively high doses of 800 international units (IU)/d were given (1 μg vitamin D = 40 IU). These inconsistencies have been extensively discussed^(^^1^^,^^19^^)^, which has led to several hypotheses that could explain the reported discrepancies. One hypothesis suggested that vitamin D was only effective in specific groups of subjects, for example, elderly living in nursing institutions. Another hypothesis was that only higher doses of vitamin D are effective, and not discriminating between different doses would mask a true effect. It was also suggested that the effect may depend on baseline and acquired circulating 25(OH)D concentrations and Ca intake. Finally, lower than expected compliance rates in the vitamin D-treated groups could have masked true effects of vitamin D.

A recent pooled analysis study by Bischoff-Ferrari *et al.*^(^^15^^)^ took several of these explanations into account, including subgroup analyses, relationship between fracture risk and baseline 25(OH)D concentrations, and actual intake of vitamin D. No effect of vitamin D treatment on fracture risk was observed using intention-to-treat analysis or treatment-dose analysis. However, using actual-intake analysis, a significant relative risk (RR) reduction of 30 % (RR 0·70; 95 % CI 0·58, 0·86) for hip fracture risk and 14 % (RR 0·86; 95 % CI 0·76, 0·96) for any non-vertebral fracture risk was observed when taking 792–2000 IU/d, whereas no effect was observed with lower vitamin D intakes^(^^15^^)^. Further analysis demonstrated that individuals having 25(OH)D concentrations of at least 61 nmol/l had a 37 % (RR 0·63; 95 % CI 0·46, 0·87) reduction of hip fracture risk and 31 % (RR 0·69; 95 % CI 0·57, 0·84) reduction of any non-vertebral fracture risk.

Individuals having baseline 25(OH)D concentrations of at least 43 nmol/l already had a significantly reduced risk for any non-vertebral fracture compared with <30 nmol/l^(^^15^^)^. This suggests that >50 nmol/l is an effective target concentration, whereas >75 nmol/l is the optimal concentration. Additional subgroup analyses within the highest actual intake group revealed that the reduction in fracture risk was consistent across groups defined by age, type of dwelling, and additional Ca intake^(^^15^^)^. Two other reviews (from the same author) investigating dose–response relationships demonstrated that the anti-fracture efficacy of vitamin D was positively correlated with acquired 25(OH)D concentrations, showing beneficial effects from about 50 nmol/l and optimal fracture reduction when concentrations of about 75–100 nmol/l were reached^(^^16^^,^^20^^)^. This dose-dependent effect was also reported in subgroup analyses in other meta-analyses, and the IOM has acknowledged that those individuals who reach at least 75 nmol/l are likely to have a reduced fracture risk^(^^1^^)^. In conclusion, it is clear that only higher vitamin D intakes (about 20 μg/d) are effective in reducing fracture risk in those individuals reaching 25(OH)D concentrations of >75 nmol/l, and it may be that this finding was missed in some previous meta-analyses due to methodological differences compared with the meta-analyses outlined above (for example, not taking actual vitamin D intake into account, or not discriminating between doses of vitamin D). It must be noted that all studies that used 20 μg/d of vitamin D also provided additional Ca to the participants.

Published dose–response studies have shown that the 25(OH)D concentration rises with approximately 1 nmol/l for each 1 μg/d of vitamin D given^(^^21^^,^^22^^)^. Thus, when baseline 25(OH)D concentrations are low, levels of 75–100 nmol/l are unlikely to be reached with 800 IU/d in the short term, which may explain the lack of anti-fracture efficiency in some studies that supplemented 800 IU/d of vitamin D in (severely) deficient patients (discussed in Bischoff-Ferrari *et al.*^(^^20^^)^). Although positive effects are observed at >50 nmol/l, we propose to define the optimal range at 75–100 nmol/l; this will allow some buffering capacity so that 25(OH)D levels will not drop below 50 nmol/l in the case of seasonal influences or temporary malabsorption problems. In the case of a severely deficient patient, we therefore propose to divide the treatment procedure into two stages: first treating the deficiency and subsequently confirming whether the optimum range has been reached, after which the maintenance dose will be prescribed; this will be described in more detail in section 3. During the winter period, intakes of 20 μg/d vitamin D will result in about 50 % of the elderly population maintaining 25(OH)D concentrations of >80 nmol/l during winter and 90–95 % of the population maintaining >50 nmol/l during winter^(^^23^^,^^24^^)^. Intakes of 30–40 μg/d would be required to ensure that 97·5 % of the population would maintain >80 nmol/l 25(OH)D^(^^24^^–^^26^^)^.

When reviewing the available literature in 2010, eight out of ten members of the International Osteoporosis Foundation Working Group concluded that 75 nmol/l should be the target value for 25(OH)D, and two members concluded that the target should be between 50 and 75 nmol/l^(^^26^^)^. These target concentrations are clearly higher than recommended by the IOM or DACH (German, Austria and Switzerland recommendation) (50 nmol/l) or the Netherlands Health Council (30 or 50 nmol/l, depending on age)^(^^1^^)^, which resulted obviously in different conclusions when reviewing the data.

We conclude that it has been sufficiently shown that ≥ 20 μg/d (800 IU/d) is effective in reducing fracture risk in ≥ 65-year-old subjects. Therefore, we recommend that individuals aged >65 years consume 20 μg/d (800 IU/d) of vitamin D. In addition, we conclude that 75–100 nmol/l is the optimal 25(OH)D range to ensure an optimal anti-fracture effect which allows some buffering capacity to maintain levels above the effective concentration of 50 nmol/l. The dose of 20 μg/d will ensure these levels for 50 % of the population, whereas 90–95 % of the population will maintain the effective range of >50 nmol/l 25(OH)D. The 50 nmol/l cut-off is also regarded effective by the Netherlands Health Council.

The acquired circulating 25(OH)D level is positively related to the anti-fracture effect of vitamin D. In cases of low baseline 25(OH)D levels, the 20 μg/d vitamin D supplementation dose appears to be insufficient in reaching the target 25(OH)D level of >50 nmol/l within a short time. Therefore, we propose that circulating 25(OH)D levels should be measured to identify and treat any pre-existing deficiency before switching to the 20 μg/d maintenance dose that is known to reduce the risk of fractures. Subsequently, additional 25(OH)D analyses after 3 and 9 months of supplementation will determine whether the optimal range of 75–100 nmol/l, or at least the effective range of >50 nmol/l, is reached. Treatment strategies for vitamin D deficiency will be discussed in section 3.

### 2.2. Data for 0- to 64-year-olds

There is sufficient evidence that vitamin D supplementation prevents rickets in young children up to 4 years old, but there are considerably fewer data available to determine optimal intakes and target concentrations for individuals aged 5–65 years^(^^3^^)^. The Netherlands Health Council recommends an intake of 10 μg/d (400 IU/d) to ensure 25(OH)D concentrations of 30 nmol/l, which is sufficient to prevent rickets in children up to 4 years old. This is well below the upper intake level of 1000–3000 IU/d as defined by the IOM for children^(^^2^^,^^3^^)^. Reference values for 25(OH)D and vitamin D intake for older children and adults are based on this observation, although effectiveness has not been studied extensively^(^^3^^)^. In addition, the relationships between vitamin D and BMD in children and adults are not clear^(^^3^^)^, but observational studies have suggested that an association exists between 25(OH)D concentrations and BMD. Based on this relationship with BMD, and the potential protective effect on colorectal cancer, it has been proposed by others that target 25(OH)D levels of 50–75 nmol/l are justified for this group^(^^20^^)^. These levels will be maintained for 50 % of the population with 10 μg/d vitamin D intake, and 20–25 μg/d would be required to ensure that 97·5 % of the general population maintains >50 nmol/l^(^^3^^,^^24^^–^^26^^)^. The 10 to 20 μg/d is in line with the Netherlands Health Council (10 μg/d) and the DACH guidelines (20 μg/d) and well below the IOM upper intake levels of 100 μg/d for adults (see below)^(^^1^^)^.

No specific recommendations have been established for pregnant and lactating women, because there are no indications that these groups have a specific higher requirement for vitamin D^(^^3^^)^. Furthermore, there are not enough data available to support different vitamin D requirements for individuals with a dark skin type. However, due to the less efficient endogenous vitamin D synthesis, supplementation is recommended for individuals with a dark skin type^(^^3^^)^.

In agreement with other recommendations for children aged 0–4 years, we propose 10 μg/d (400 IU) and reference values of 30 nmol/l 25(OH)D.

For ages 5–64 years, we conclude that there is a lack of solid evidence that supports well-defined recommendations for vitamin D supplementation. Although BMD is an indirect measure of bone health, we consider it to be beneficial to optimise BMD throughout life. Although solid data from intervention studies are lacking, observational studies have reported a positive association between 25(OH)D level and BMD, suggesting an optimal concentration range of 50–75 nmol/l. We therefore assume that 50–75 nmol/l is the optimal range for ages 5–64 years. At least 10 μg/d (400 IU/d) is required to ensure 25(OH)D concentrations of >50 nmol/l for 50 % of the population, and preferably 20 μg/d (800 IU/d) is recommended for this 25(OH)D level for 97·5 % of the population. No special requirements are made for pregnant or lactating women, or individuals with a dark skin type. Similar to ages > 65 years, we propose to measure 25(OH)D levels at baseline to identify and treat any deficiency, and to confirm after 3 and 9 months that optimal levels are reached.

## 3. Regimens for treating vitamin D deficiency and maintaining optimal levels

There are indications that vitamin D intakes that are described in the current literature to maintain adequate 25(OH)D levels will not be sufficient to correct a deficient state. Therefore, a main aim of NutriProfiel is to provide a recommendation to treat vitamin deficiency before maintaining optimal vitamin status. Although many protocols for the treatment of deficiency are being used in healthcare practice, the evidence-based foundation of these protocols is often lacking. This section describes the relationship between vitamin D intake and status, introduces a loading protocol to treat deficiency in adults, and provides recommendations for the maintenance dose regimen once the optimal 25(OH)D levels are reached. Also the influence of UV exposure on vitamin D status is discussed.

### 3.1. Maintaining and correcting vitamin D status

Considerable efforts have been made to investigate the effect of different vitamin D doses on circulating 25(OH)D concentrations. To date, the published dose–response studies have revealed that 1 μg/d of vitamin D is required for each 1 nmol/l increase of 25(OH)D^(^^6^^,^^21^^,^^22^^)^. Cashman *et al.* calculated that about 10 μg/d of vitamin D will result in only 50 % of the population reaching 25(OH)D concentrations of >50 nmol/l^(^^23^^–^^25^^)^. In fact, doses of at least 20 μg/d would be needed to ensure that 50 % of the population would maintain >80 nmol/l 25(OH)D, and 97·5 % of the adult population would maintain 25(OH)D levels of >50 nmol/l during winter (following summer-time levels of about 60–70 nmol/l). This intake level, which is two times higher than the Health Council recommends for individuals aged <70 years, is considered to be insufficient to raise 25(OH)D levels above 75 nmol/l in most of the individuals, underlining the need for loading protocols before maintenance dosing^(^^25^^,^^27^^,^^28^^)^. It has also been described that elderly respond less to supplementation due to lower baseline levels^(^^28^^)^. All together, this provides strong arguments to start with a loading dose to ensure that optimal 25(OH)D levels are quickly reached before switching to the maintenance dose.

The usefulness of loading protocols has been discussed before^(^^28^^)^. Van Groningen *et al.*^(^^27^^)^ undertook a dose-escalation study using subjects with a wide variety in age (18–88 years old), baseline 25(OH)D levels (range: <10 to 47 nmol/l) and body weight to determine an optimal loading-dose protocol. By measuring 25(OH)D before and after supplementation using different loading regimens, they were able to demonstrate that baseline levels and dose per kg body weight are the most important factors influencing the dose–response curve. In contrast, age, sex, BMI, body length or season did not significantly affect the rise in circulating 25(OH)D levels^(^^27^^)^. A simplified relationship was extracted, being: Δ25(OH)D = 0·025 × dose per kg body weight (in IU). A similar finding was presented by Drincic *et al.*^(^^29^^)^, who showed that the response to oral vitamin D depended on body size. In addition, they recommend that the loading dose should be given in portions of 25000 IU per week, and that the formula is not valid for individuals <18 years of age, having a body weight >125 kg, or having a BMI of >40 kg/m^2^. To ensure that target 25(OH)D levels are reached, measurements at 3 and 9 months after the start of supplementation should be performed.

For deficiency treatment in children <18 years old, no specific loading regimens have been reported. Therefore, we propose to dose the generally accepted 1 μg/d for any 1 nmol/l 25(OH)D increase that is required to reach optimal levels with the understanding that the accepted upper daily intake levels that have been set for children (see Table 1) should never be exceeded. To ensure that target 25(OH)D levels are reached, measurements at 3 and 9 months after the start of supplementation should be performed. It is good to note that this advice is different from what is described in the Netherlands ‘Farmacotherapeutisch Kompas’.

**Table 1.** Summary of NutriProfiel recommendations for circulating 25-hydroxyvitamin D (25(OH)D) concentrations and vitamin D intake, split per age group

	Circulating 25(OH)D (nmol/l)			Vitamin D_3_ intake		
Age group	Deficiency	Sufficiency	Optimal	μg/d	IU/d*	Upper daily intake (IU/d*)
0–6 months	< 20	20–30	30–50	10	400	1000
6–12 months	< 20	20–30	30–50	10	400	1500
1–4 years	< 20	20–30	30–50	10	400	2500†
5–8 years	< 30	30–50	50–75	10–20	400–800	3000
8–64 years	< 30	30–50	50–75	10–20	400–800	4000
> 65 years	< 50	50–75	75–100	20	800	4000

* 1 μg vitamin D = 40 IU.

† Upper daily intake for 4-year-olds is 3000 IU.

It is concluded that vitamin D loading is needed to ensure that target 25(OH)D levels are quickly reached before following the recommended maintenance dose. First, serum 25(OH)D concentration should be determined in all subjects. When subjects are already in the optimal range, no loading is required and subjects can directly follow the recommended dose (see sections 2 and 3·2). When a subject is deficient, the Van Groningen protocol^(^^27^^)^ is suitable to resolve this for all adults >18 years old. For children <18 years old, deficiency can be treated by loading with 1 μg/d for any desired 1 nmol/l increase in 25(OH)D. As already mentioned above, circulating 25(OH)D levels should be analysed after 3 and 9 months to verify that optimal 25(OH)D concentrations are reached.

### 3.2. Administration of maintenance dose

Once the optimal 25(OH)D levels are reached, the maintenance vitamin D dose can be supplemented on a daily, weekly, monthly or yearly basis. The half-life of vitamin D_3_ varies between 3 and 6 weeks^(^^30^^)^ so it can be expected that a yearly bolus supplementation will not ensure stable circulating 25(OH)D levels throughout the year. In fact, a yearly bolus regimen is not recommended in the literature due to lack of efficacy and suboptimal gastrointestinal absorption. As a rule of thumb, a drug should be administered at least once during its half-life. This means that protocols that describe supplementation once every 3 or 4 months are clearly inadequate. Based on the pharmacokinetic profile, daily or weekly administration of vitamin D is likely to result in the most stable 25(OH)D concentrations, but there are no data available that support a choice between the two options based on clinical outcomes. For convenience, we recommend to ensure vitamin D intake on a daily basis. If this would not be feasible for any reason, then weekly administration is a suitable second choice.

It is recommended to administer the optimal vitamin D dose (see section 2) preferably on a daily basis, and to consider weekly administration when daily administration is not feasible.

### 3.3. Interaction with UV exposure/endogenous vitamin D synthesis and use of supplements

Exposure to UV light drives the endogenous synthesis of vitamin D in the skin, and this contributes to the vitamin D status. It is, however, very difficult to make precise estimations of the amounts that are synthesised at the population level due to the fact that skin synthesis depends on a number of factors that are highly variable between individuals, such as skin surface area exposed to sun, duration of sun exposure, use of sun creams, time of day/year and efficiency of the skin to synthesise vitamin D^(^^3^^)^. Precise data are lacking for the Netherlands, but the variation in vitamin D synthesis is estimated to range between >10 μg/d in summer to virtually no endogenous synthesis during winter, and depends on many factors. The Health Council of the Netherlands acknowledges the lack of solid individual data to make reliable quantifications of the contribution of UV exposure to the vitamin D status, but recommended that individuals with a light skin type who get sufficient exposure to sun light produce enough endogenous vitamin D to meet the requirements and are therefore not advised to use vitamin D supplements. Sufficient sun exposure is defined as spending 15–30 min outdoor between 11.00 and 15.00 hours from March to November, with head and hand skin areas exposed to the sun; based on indirect calculations, this amount of sun exposure is expected to be sufficient to synthesise on average 6–7 μg/d throughout the year. The Health Council has proposed that individuals with a dark skin type, or those who do not get sufficient exposure to UV light, should use vitamin D supplements in order to meet the requirements^(^^3^^)^. In addition, all women between 50 and 70 years of age are advised to take a 10 μg/d supplement, and all adults over 70 years of age are advised to take a 20 μg/d supplement to ensure that the vitamin D requirements are met^(^^3^^)^.

We conclude that the inter-individual and seasonal variation in sun exposure and hence endogenous vitamin D synthesis is quite large. The recommended vitamin D intake should be sufficient throughout the whole year to maintain optimal levels. The need to use supplements depends on skin type and UV exposure. Individuals with a dark skin type, or individuals that do not get sufficient amounts of UV light exposure, should use supplements to meet the vitamin D requirements and maintain their vitamin D levels. In addition, women over 50 years are advised to use 10 μg/d using a supplement, and all adults >70 years are advised to use a 20 μg/d supplement.

## 4. Safety of vitamin D supplementation

### 4.1. Toxicology of vitamin D

Safety of vitamin D has received much attention in the past, but there are still many uncertainties. The primary consequence of vitamin D intoxication is the development of hypercalcaemia, which could lead to adverse effects such as vomiting, pain, fever, anorexia and weight loss. Information about vitamin D intoxication is limited to anecdotal evidence, with extremely high intakes of at least 1250 μg/d or extremely high UV exposure causing classical signs of toxicity^(^^31^^,^^32^^)^. So far, most controlled experiments supplementing about 10 to about 1000 μg/d of vitamin D (including additional Ca in some cases) did not report any adverse effects or hypercalcaemia^(^^28^^,^^31^^,^^32^^)^. There seems to be consensus that a prolonged daily intake of 250 μg/d (10000 IU/d) does not cause adverse effects^(^^1^^,^^3^^)^. The IOM has converted this with a safety factor of 2·5 into 100 μg/d (4000 IU/d) as a safe upper limit of intake for adults, and defined 1000–3000 IU for children up to 8 years^(^^1^^,^^2^^)^. Circulating 25(OH)D levels of up to 220 nmol/l are considered to be safe because these levels correspond with prolonged intake of 250 μg/d, for which no change in circulating Ca levels were observed^(^^28^^,^^32^^)^. In addition, 25(OH)D concentrations up to 140 nmol/l were not associated with an increased all-cause mortality risk, whereas concentrations <75 nmol/l showed an increased risk for all-cause mortality^(^^33^^)^. Another recent meta-analysis demonstrated that serum 25(OH)D concentrations below 75 nmol/l were associated with higher all-cause mortality compared with concentrations higher than 75 nmol/l^(^^34^^)^. These studies suggest that a J-shaped relationship exists between circulating 25(OH)D concentrations and all-cause mortality. This finding supports the previous notion that 25(OH)D concentrations up to 220 nmol/l are safe, and that concentrations higher than 75 nmol/l may result in beneficial health effects.

High vitamin D intake combined with high Ca intake may increase CVD risk or the formation of renal stones, which could be explained by a high use of self-selected supplements^(^^1^^,^^32^^)^, underlining the need for careful well-founded dietary advice. In conclusion, the IOM considers vitamin D intakes of up to 100 μg/d safe for the general population. No specific guidelines for pregnant or lactating women, infants, children, elderly or specific diseases were found, except some specific warnings for individuals with high Ca intake (see below).

Following the IOM's recommendations, vitamin D supplementation up to 100 μg/d ( = 4000 IU/d) for adults, and serum 25(OH)D levels up to 220 nmol/l, can be considered safe. Different upper intake levels, as set by the IOM, should be applied for children, being 25 μg/d (1000 IU/d) for 0–6 months, 37·5 μg/d (1500 IU/d) for 6–12 months, 65·5 μg/d (2500 IU/d) for 1–3 years, and 75 μg/d (3000 IU/d) for 4–8 years of age. Caution should be taken when Ca is supplemented in addition to vitamin D.

### 4.2. Effect of calcium in combination with vitamin D

Ca is important for bone health, and the effect and safety of additional Ca intake on bone health in the context of vitamin D supplementation is heavily debated. As indicated above, a pooled analysis demonstrated that only the highest vitamin D supplementation doses would reduce fracture risk independent of Ca intake, which is supported by findings that with sufficient Ca and vitamin D intake, a higher Ca intake does not improve bone health^(^^30^^)^. A meta-analysis revealed that high Ca intakes (about 500–1000 mg/d from supplements) may be related to cardiovascular events and kidney stones in subjects that already had about 800 mg/d Ca intake^(^^35^^)^; high Ca intakes from supplements should therefore be avoided. Recommendations for adequate Ca intake in the Netherlands vary between 1000 and 1200 mg per d^(^^36^^)^. The exact interaction between (supplemental) Ca, vitamin D and adverse health effects is still a matter of ongoing research. To the best of our knowledge, we have not found any data that suggest that the proposed optimum intakes of 20–25 μg/d vitamin D, in combination with a total Ca intake of 1000–1200 mg/d, will result in adverse health effects. There are indications that individuals with a Turkish, Moroccan or Surinamese background have inadequate Ca intakes^(^^36^^)^; in these cases it may be useful to administer supplemental Ca although care must be taken to avoid hypercalcaemia. It must be noted that the effectiveness of such a ‘personalised’ strategy (for example, only supplementing Ca in at-risk groups) remains to be demonstrated.

We conclude that supplemental Ca intake has no beneficial effect on fracture risk when vitamin D intake is already sufficient. Intakes of Ca in excess of dietary recommendations might cause cardiovascular events or kidney problems. Therefore, we do not recommend Ca supplementation in addition to vitamin D when Ca intake is already between 1000 and 1200 mg/d. Additional Ca can be considered when dietary Ca intake is inadequate. Milk, dairy products and cheese are foods that contain Ca and could be used to increase Ca intake.

## 5. Differences compared with the Netherlands Health Council advice on vitamin D

The NutriProfiel recommendations for intakes of vitamin D and target concentrations of 25(OH)D are different from recommendations made in 2012 by the Netherlands Health Council^(^^3^^)^. Major discrepancies are the difference between minimum and optimum vitamin D intakes, and the inclusion of a deficiency treatment strategy in the NutriProfiel advice, which will be outlined below.

The Health Council made recommendations to ensure ‘minimum’ levels of 25(OH)D to prevent bone disease, for which they have carefully weighed the available data before formulating their recommendations. The purpose of NutriProfiel is to provide a comprehensive approach of vitamin D status testing, treatment of deficiency, and maintenance of optimal 25(OH)D levels, and for this purpose we have examined the available scientific literature. There is a considerable amount of data from RCT that does support the significance of >50 nmol/l, and this is discussed elsewhere^(^^6^^–^^8^^,^^15^^,^^16^^,^^20^^)^. In addition, meta-analyses of RCT investigating bone health have supported the notion that there are additional health benefits when concentrations of >75 nmol/l are reached in elderly^(^^15^^,^^16^^,^^20^^)^. Since safety does not seem to be an issue, and levels of 75 nmol/l are encountered on a routine basis in the Netherlands^(^^6^^)^, we conclude that there is sufficient evidence to define >50 nmol/l and >75 nmol/l 25(OH)D as the optimum concentrations for 5–64 and >65 years of age, respectively, for which there are no safety concerns. Regarding the vitamin D intake for 5–64 years old, 10 μg/d and preferably 20 μg/d are based on dose-finding studies that revealed that this intake is sufficient to maintain 25(OH)D levels at >50 nmol/l in about 97·5 % of the adult population. For >65 years of age, the 20 μg/d (1000 IU/d) dose is chosen based on meta-analysis that revealed that >20 μg/d (800 IU/d) was effective in preventing hip fractures. It is good to note that the NutriProfiel recommendations are largely in line with DACH guidelines, and that the recommended intakes are well below internationally accepted upper intake levels.

In addition to most recommendation-setting bodies that only provide recommendations to maintain vitamin D status, the NutriProfiel recommendations also contain a strategy to diagnose and treat deficiency. It can be expected that doses advised by the Netherlands Health Council (10 or 20 μg vitamin D/d) will not correct a severe deficiency, a condition that is often encountered in a clinical setting, especially during winter. In addition, ensuring that all subjects reach the optimum 25(OH)D levels quickly is likely to improve long-term health outcomes. This is supported by meta-analyses, that have revealed that the anti-fracture efficacy correlated with the acquired circulating 25(OH)D concentrations after supplementation. In addition, it has been speculated that lack of efficacy of vitamin D in certain RCT can be explained by the fact that these studies were performed in deficient subjects. Therefore, it is likely that using a loading regimen to treat a vitamin D deficiency will contribute to the long-term health effects of vitamin D.

## 6. Summary: NutriProfiel advice for dietary intake, plasma/serum concentrations, and dosing regimens for vitamin D

In view of the findings outlined above, we summarise our recommendations in Table 1. We advise using different levels to define deficiency, sufficiency and optimal concentrations of 25(OH)D for different age groups. Deficiency means that there is insufficient protection against osteomalacia and fractures. Deficiency levels are obtained from previous recommendations^(^^2^^,^^3^^)^. An optimal concentration means that there is adequate protection against chronic diseases or conditions with a progressive pathophysiology (for example, fracture risk), and sufficiency includes concentrations between deficiency and optimal 25(OH)D concentrations. Both are derived from the data outlined above. Upper daily intake levels are derived from IOM recommendations.

For children aged 0–4 years, 10 μg/d (400 IU/d) would be sufficient, with an optimal 25(OH)D concentration of 30–50 nmol/l.

For 5–64 years 10–20 μg/d (400–800 IU/d) with an optimal 25(OH)D concentration range of 50–75 nmol/l are recommended.

For >65 years a daily intake of 20 μg/d (800 IU/d) and an optimal 25(OH)D concentration range of 75–100 nmol/ml are recommended.

No special recommendations are made for pregnant or lactating women, or individuals with a dark skin type.

A loading regimen according to Van Groningen *et al.*^(^^27^^)^ can be followed to treat deficiency in adults >18 years old, after which the recommended intake should be enough to maintain serum 25(OH)D levels. The optimal 25(OH)D concentration is the target, and loading is required when the 25(OH)D concentration is below the optimal range. Measurements of 25(OH)D after 3 and 9 months will determine whether reference 25(OH)D concentrations are reached and if dose adjustment is needed. For children <18 years old, deficiency is treated by loading with 1 μg/d for every 1 nmol/l increase in 25(OH)D that is required to reach the optimal range, after which the maintenance dose is recommended.

Safety should not be an issue with the recommended intakes, and 25(OH)D levels should never exceed 220 nmol/l. Ca should only be supplemented when Ca intake is below recommended values; dietary counselling is advised in these circumstances. Based on these doses, we do not expect hypercalcaemia to occur. Serum Ca levels should only be measured in the unlikely event when hypercalcaemia is suspected.

## Appendix. Workshop participants

### From the Division of Human Nutrition, Wageningen University (Wageningen, the Netherlands)

Evelien Backx (PhD candidate), Fränzel van Duijnhoven (researcher), Lisette de Groot (professor in Nutrition and Ageing; speaker), Ellen Kampman (professor in Nutrition and Cancer), Nicole de Roos (assistant professor) and Michael Tieland (researcher).

### From Gelderse Vallei Hospital (Ede, the Netherlands)

Hans-Peter Bootsma (hospital pharmacist), Ellen Dutmer (rheumatologist), Geert Feith (nephrologist), Inez Jans (dietitian), Jacqueline Klein Gunnewiek (clinical chemist; speaker), André Janse (geriatrician), Wout van Orten-Luiten (scientist), Emmelyne Vasse (dietitian) and Ben Witteman (gastroenterologist).

### From General Practice Bennekom (Bennekom, the Netherlands)

Elvira Schouten (general practitioner).
